# Supplementary material for: A network-level test of the role of the co-activated default mode network in episodic recall and social cognition
Source: Cortex. 2023 Aug;165:141–59. doi: 10.1016/j.cortex.2022.12.016 (PMC10284259; doi:10.1016/j.cortex.2022.12.016)
Supplement: Multimedia component 1 [file mmc1.docx]

A Network-level Test of the Role of the Co-activated Default Mode Network in Episodic Recall and Social Cognition

Rebecca L. Jackson, Gina F. Humphreys, Grace E. Rice, Richard J. Binney & Matthew A. Lambon Ralph

**Supplementary Materials***
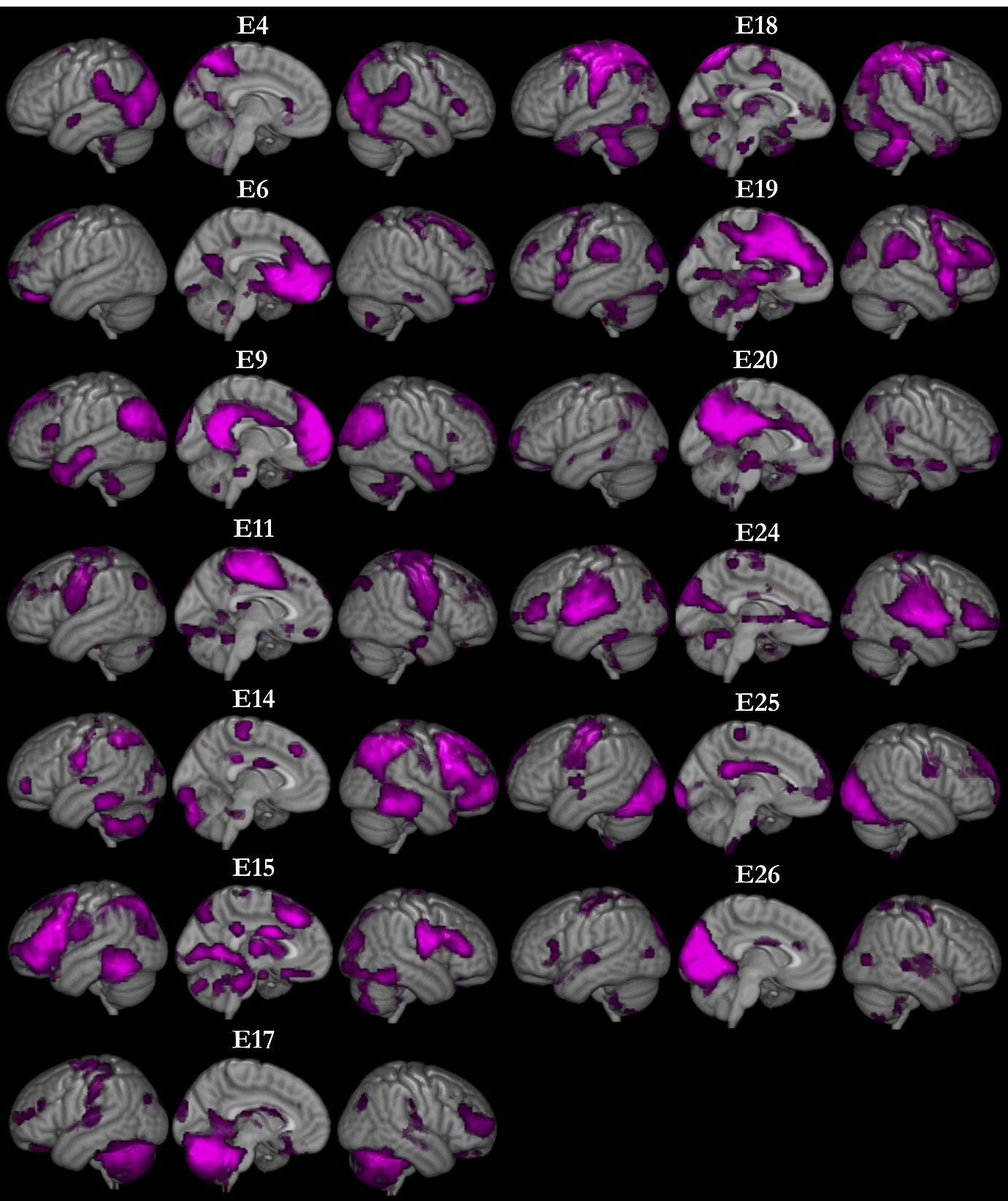
*

*Supplementary Figure 1.* Each non-artefactual component identified in the *Episodic Task* dataset is shown in violet with its corresponding label.


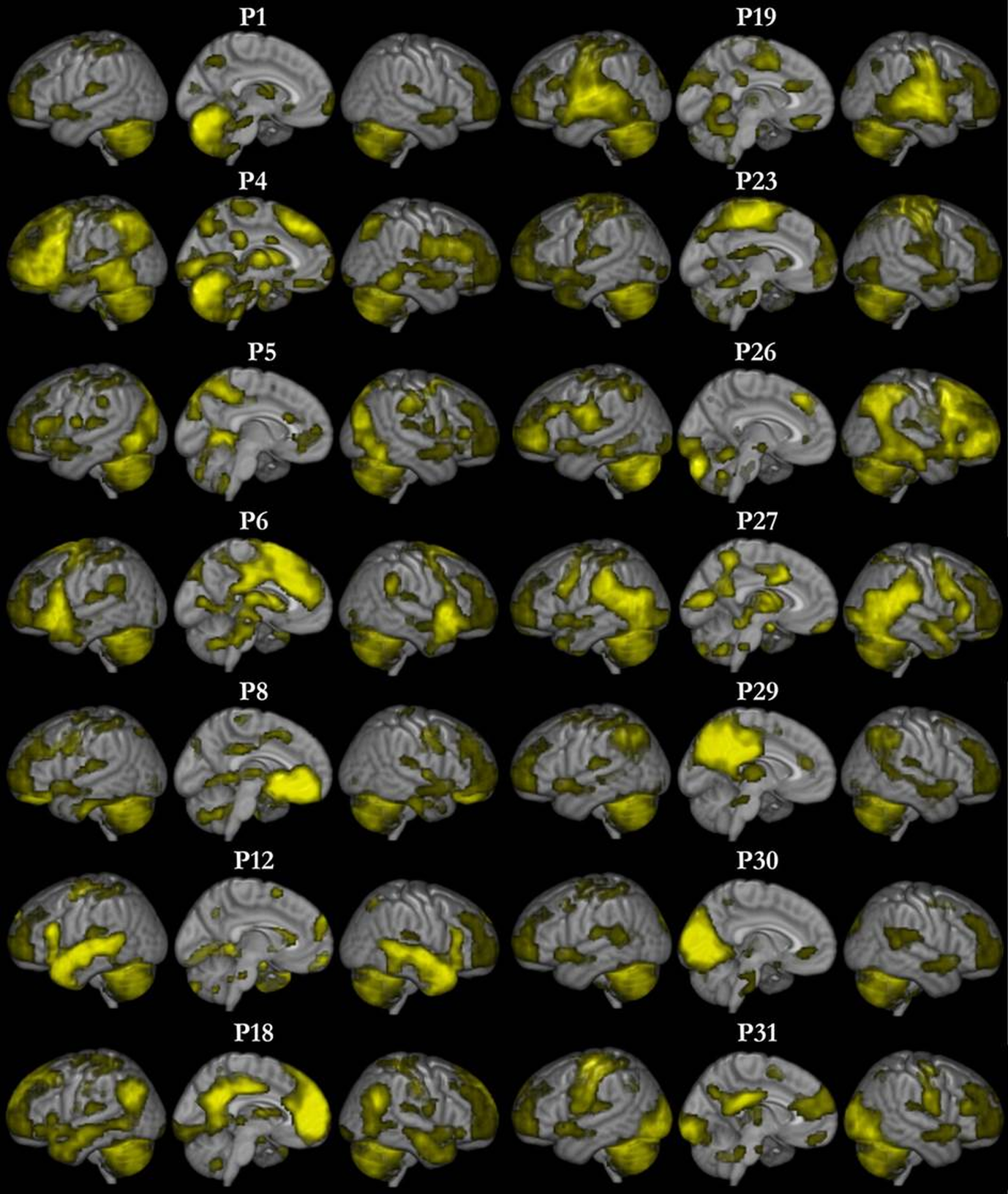


*Supplementary Figure 2.* Each non-artefactual component identified in the *Social - Person Knowledge Task* dataset is shown in yellow with its corresponding label.


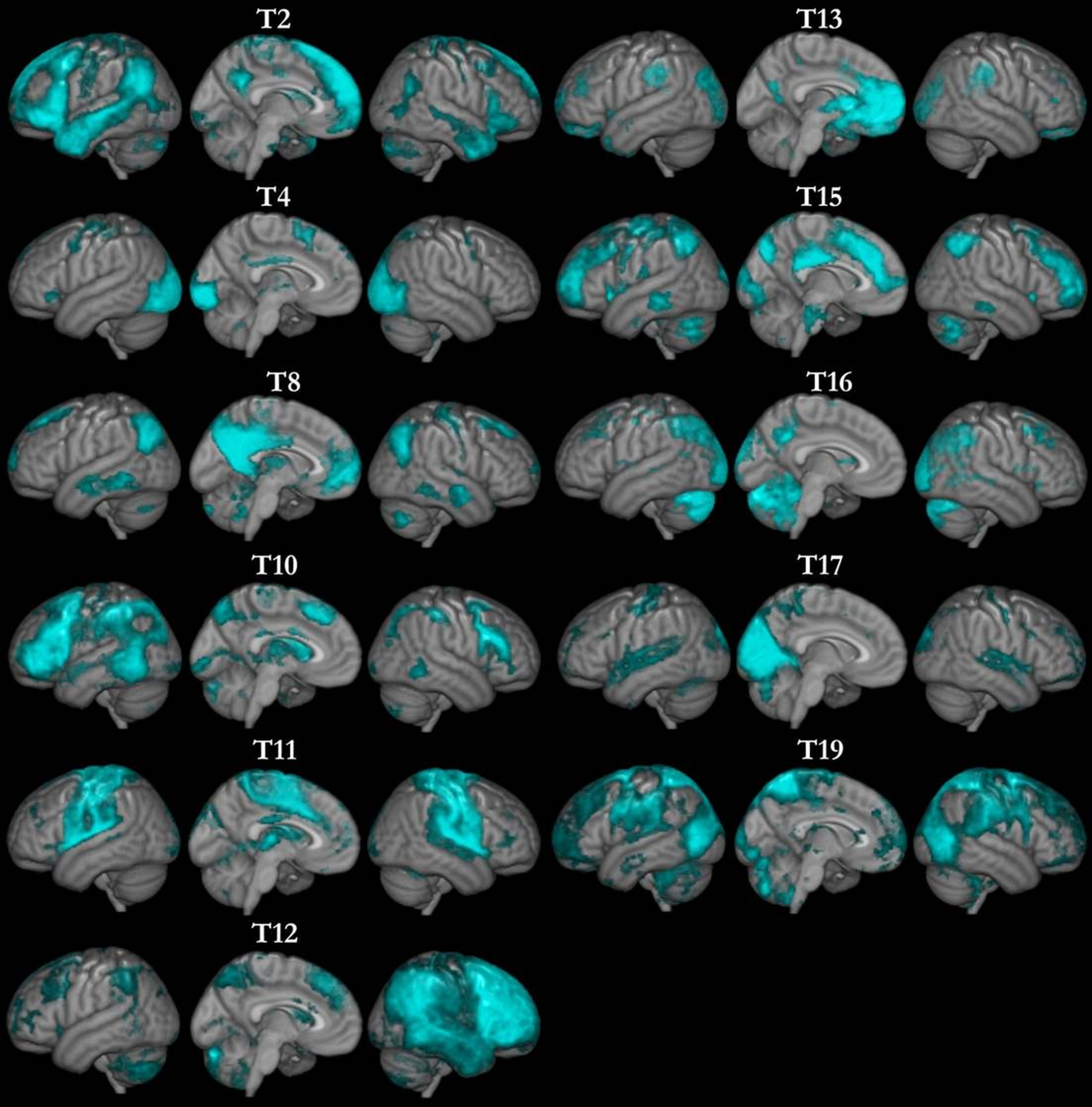


*Supplementary Figure 3.* Each non-artefactual component identified in the *Social - Theory of Mind Task* dataset is shown in cyan with its corresponding label.

*
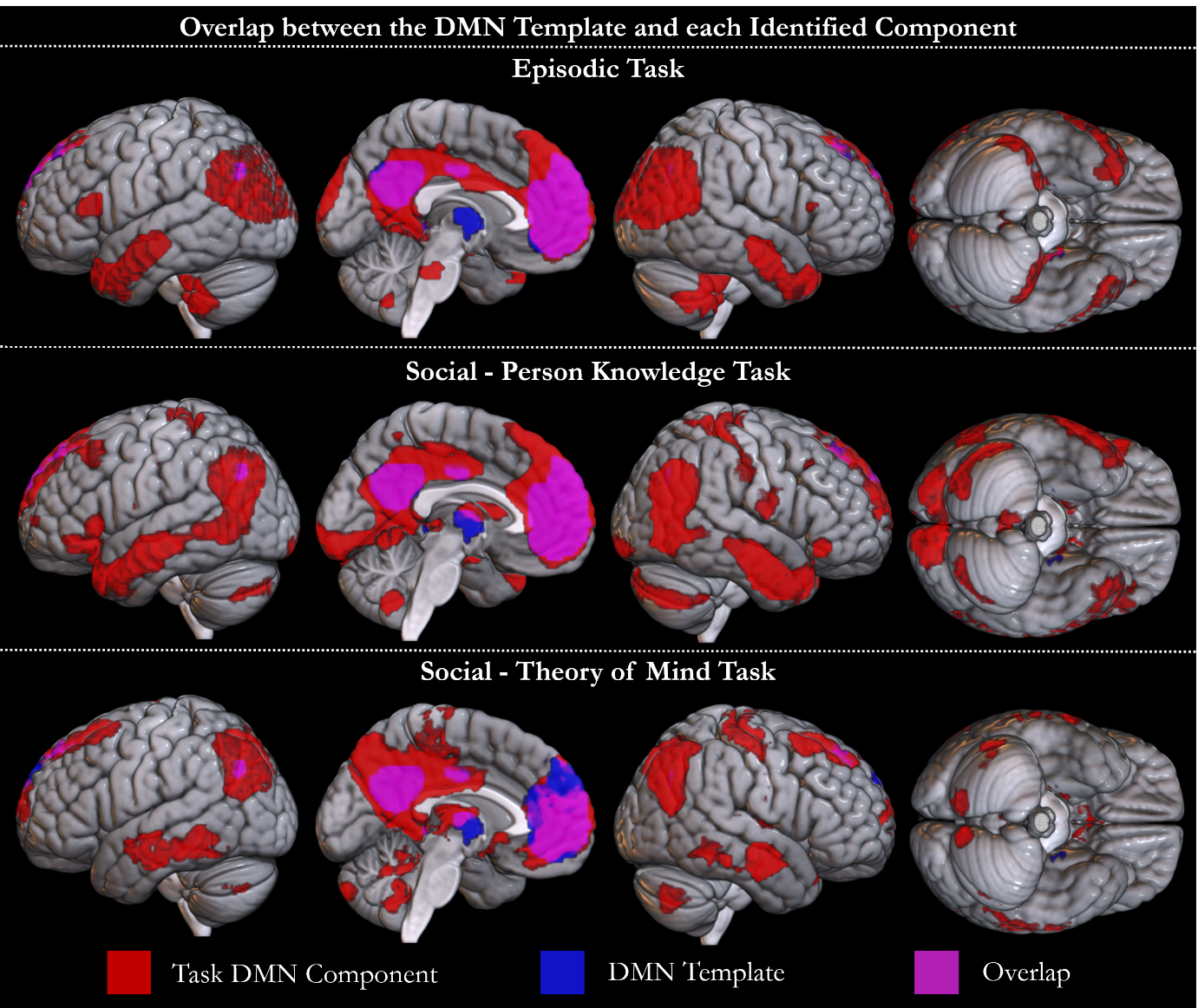
*

*Supplementary Figure 4.* The spatial overlap between the *a priori* template obtained from Shirer et al., (2012) and the component identified to be the DMN in each task is shown. The components identified in the *Episodic Task* (E9), *Social - Person Knowledge Task* (P18) and *Social - Theory of Mind Task* (T8) are shown in red. The template is shown in blue. Overlap is shown in violet.

*
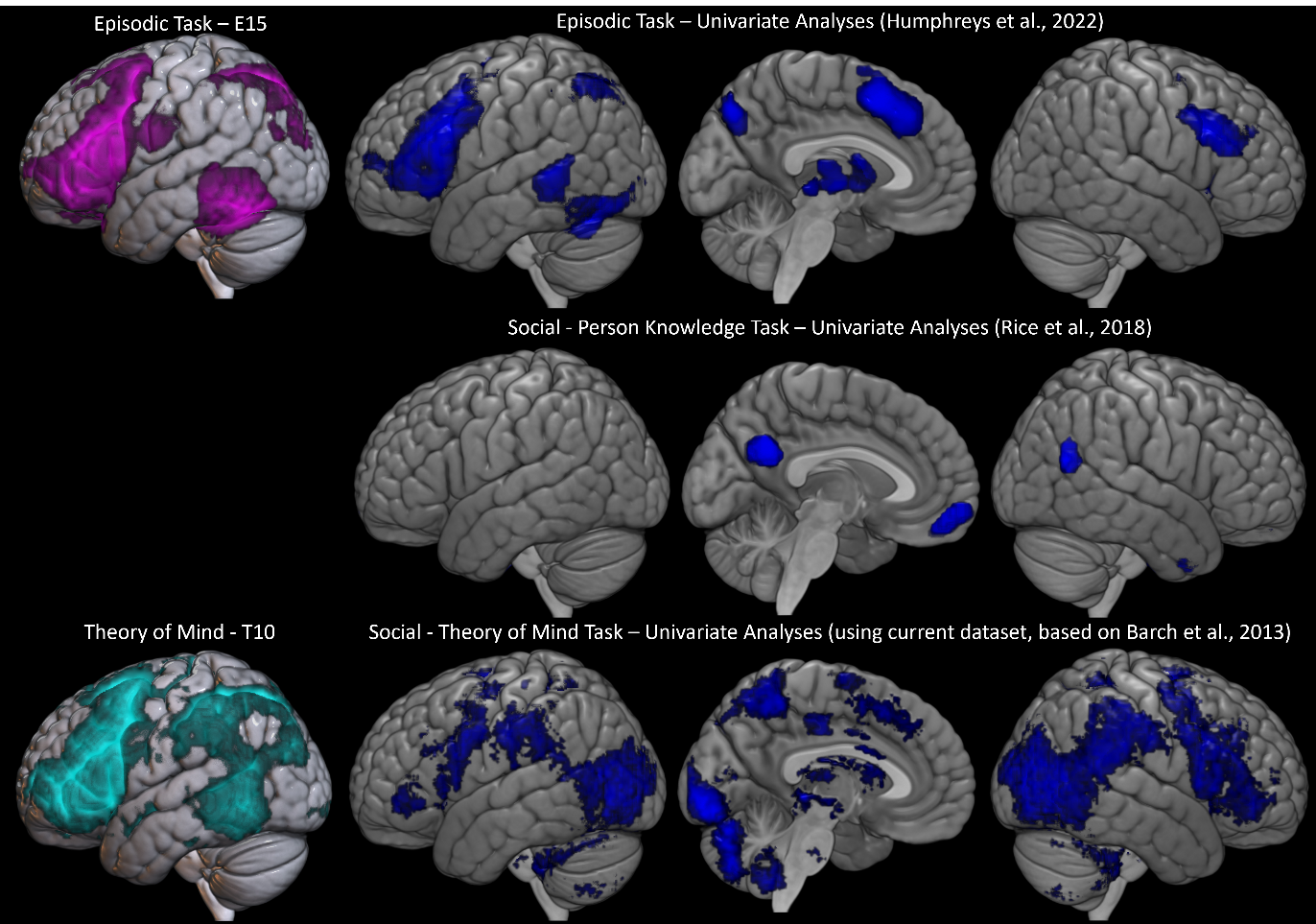
Supplementary Figure 5.* The univariate contrast maps from each task (clusters displayed are significant using FWE-correction and a critical cluster level of 0.05). The components demonstrating significant task differences are shown alongside the relevant univariate contrasts for comparison. Note however, that network-level and region-level assessments are expected to differ. Top: the episodic>control contrast from Humphreys et al. (2022) is presented alongside the component showing the strongest episodic>control effect (although note the involvement of additional components in this task). Middle: the social>non-social contrast in the Person Knowledge task performed by Rice et al., (2018). This is shown alone as no components were significantly engaged for social>non-social semantics. However, note that two components did show significant involvement in both social and non-social semantics. Bottom: a univariate contrast for the social>non-social condition in the Theory of Mind task performed using the current dataset. This is a replication of the analysis in Barch et al., (2013) using data from the specific HCP participants included in the current study. It is shown with the single component which showed greater engagement for the social than non-social condition.
